# Liver-Derived Cell Transfection Model Efficacy for HBV Genotype B Replication/Transcription Is Determined by Complex Host Transcription Factor Network

**DOI:** 10.3390/v13030524

**Published:** 2021-03-22

**Authors:** Roxanne Hui-Heng Chong, Atefeh Khakpoor, Theresa May-Chin Tan, Seng-Gee Lim, Guan-Huei Lee

**Affiliations:** 1 Department of Medicine, Yong Loo Lin School of Medicine, National University of Singapore, Singapore 117549, Singapore; huihengc@hotmail.com (R.H.-H.C.); mdcatef@nus.edu.sg (A.K.); mdclimsg@nus.edu.sg (S.-G.L.); 2 Department of Biochemistry, Yong Loo Lin School of Medicine, National University of Singapore, Singapore 117597, Singapore; bchtant@nus.edu.sg; 3 Department of Medicine, National University Hospital, Singapore 119074, Singapore

**Keywords:** hepatitis B virus, transcription factor, transfection model, genotype B

## Abstract

Background: Interaction between host transcription factors (TFs) and the viral genome is fundamental for hepatitis B virus (HBV) gene expression regulation. Additionally, the distinct interaction of the TFs’ network with the HBV genome determines the regulatory effect outcome. Hence, different HBV genotypes and their variants may display different viral replication/transcription regulation. Due to the lack of an efficient infection model suitable for all HBV genotypes, the hepatoma cell transfection model is primarily used in studies involving non-D HBV genotypes and variants. Methods: We explored the transcriptome profile of host TFs with a regulatory effect on HBV in eight liver-derived cell lines in comparison with primary human hepatocytes (PHH). We further analyzed the suitability of these models in supporting HBV genotype B replication/transcription. Results: Among studied models, HC-04, as a result of the close similarity of TFs transcriptome profile to PHH and the interaction of specific TFs including HNF4α and PPARα, showed the highest efficiency in regard to viral replication and antigen production. The absence of TFs expression in L02 transfection model resulted in its inefficiency in HBV replication/transcription. Conclusion: These observations help to better design studies on regulatory mechanisms involving non-D HBV genotypes and variants’ gene expression and the development of more efficient therapeutical approaches.

## 1. Introduction

Hepatitis B virus (HBV), the causative agent for chronic HBV infection (CHB), is a major global health risk factor [1]. Similar to other viruses (HPV, EBV, etc.) [2,3], HBV gene expression regulation is an interplay between viral genome and host factors. Accumulating evidence suggests that precise recruitment of host transcription factors (TFs) to distinct regulatory sites on viral covalently closed circular DNA (cccDNA) leads to viral gene expression regulation [4,5,6,7]. HBV genome cloning and mapping of viral transcripts have led to a comprehensive understanding of the promoters (pre-core/core, X, pre-S1, and pre-S2/S promoters) and enhancers (Enh) I and II regulatory sites. These sites control the synthesis of viral transcripts including the replication intermediate, pre-genomic RNA (pg-RNA) in cooperation with host TFs [1,5,8,9].

Host TFs tropism is either restricted to hepatocytes (liver enriched TFs) or expressed in other cell types, which are categorized as ubiquitous TFs [5,7]. Past studies have shown that liver enriched TFs, specifically, the hepatocyte nuclear factor family (HNF) play a fundamental role in HBV replication/transcription regulation [5,6,7,8]. Enhanced activities from preS1, preS2/S, core promoter, and Enh- II as a result of binding of liver enriched TFs, including HNF1α, HNF3α and HNF4α were observed using hepatoma cells [9,10,11]. Apart from liver enriched TFs, several ubiquitous TFs do play crucial roles in HBV replication/transcription [5]. SP-1 and NF1 binding to the S promoter are essential for promoter optimal activity [6,12]. Studies by Ori et al. have established the positive regulatory role of NF1 on pg-RNA synthesis through binding to Enh-I [10,13].

HBV gene expression regulation is modulated by the distinct site of interaction and the expression level of TFs. Hence, possible alterations in the interaction sites among HBV genotypes and their variants as a result of mutations may lead to different regulatory effects of TFs on each genotype [5,6,7,8]. Despite the discovery of HBV entry receptor [14] and establishment of a highly efficient in vitro infection model supporting HBV genotype D [15], studies on non-D HBV genotypes and variants are hampered by the lack of an existing high titer viral source for efficient infection in an existing in-vitro model. Hence, liver-derived cell transfection models are the preferred approach in these studies.

Here, we investigate the transcriptome and proteome profile of selected liver enriched and ubiquitous TFs with regulatory roles in HBV gene expression, across eight established human liver-derived cell lines in comparison with primary human hepatocytes (PHH). We further studied how host TFs expression regulates HBV genotype B replication and gene expression in transfection models either with hepatocellular carcinoma-derived lines (HCC) (HepG2, HepG2-hNTCP, Huh-7, HuH-6, and JHH-4) or immortalized cells of human hepatocyte (L02, HC-04, and THLE-2) origin.

## 2. Materials and Methods

### 2.1. Cell Culture

PHH batches from 3 different donors (Gentest^TM^ plateable cryohepatocytes, BD Biosciences, USA) were negative for human immunodeficiency virus (HIV), HBV, and hepatitis C virus (HCV). Cells were cultured on BioCoat collagen I plate (Corning) using Gentest^TM^ Plating Medium supplemented with 10% fetal bovine serum (FBS).

HCC cell lines (HepG2, Huh-7, HuH-6, JHH-4 [16,17,18,19], HepG2-hNTCP [15], HepG2.2.15) and immortalized human hepatocyte lines (L02, HC-04, THLE-2) were incubated at 37 °C under 5% CO_2_ in media as described in Table 1.

### 2.2. TFs Transcriptome Analysis

Cells were harvested at 48 h post-seeding and subjected to:

1. RNA extraction and cDNA synthesis. Total RNA was extracted using RNeasy Mini Kit (Qiagen, Hilden, Germany) and lysates were treated with RNase-free DNase I (New England Biolabs) before proceeding to total RNA reverse transcription with oligo(dT)_20_ SuperScript^®^ III First-Strand Synthesis System (Invitrogen, Thermo Fisher Scientific) following the manufacturer’s instructions.
2. Real-time PCR. Real-time PCR was performed using the QuantiTect SYBR^®^ Green PCR Kit (Qiagen) on the ABI prism 7000 sequence detection system or QuantStudio 6 Flex Real-Time PCR System (Applied Biosystems, Thermo Fisher Scientific). Table 2 summarizes the primer sequences used. Endogenous β-actin transcript level [21] was used for normalization. Relative mRNA expression levels were calculated using ∆∆Ct method. The hierarchical clustering analysis was based on the mean of log2-transformed data using the complete linkage method with Euclidean distance measure. Heatmap was generated using R software.

### 2.3. TFs Proteome Analysis

Forty-eight hours post-seeding, nuclear protein was extracted and subjected to SDS polyacrylamide gel electrophoresis followed by transfer to nitrocellulose membranes. Membranes were blocked and probed with primary antibodies (Table 3) followed by horseradish peroxidase-conjugated secondary antibodies probing, goat anti-mouse IgG, goat anti-rabbit IgG (Thermo Fisher Scientific, MA, USA), and donkey anti-goat IgG (Santa Cruz Biotechnology, TX, USA). Protein bands were visualized using enhanced chemiluminescence (ECL) by Advansta ECL kit. Quantitative analysis of the signal intensity was performed using Quantity One 1-D Analysis Software (Bio-Rad Laboratories, CA, USA).

### 2.4. Transient Transfection of Full-Length HBV DNA Genome

A full-length HBV clone was generated from a genotype B HBeAg-negative reactivation serum sample according to the method described by Günther et al. [22]. Linear HBV monomers were prepared from the plasmid by BspQI (New England Biolabs, MA, USA) digestion and gel purification using QIAquick Gel Extraction Kit (Qiagen, Hilden, Germany). Transfection was carried out using Effectene (Qiagen, Hilden, Germany) or X-tremeGENE HP DNA Transfection Reagent (Roche, Switzerland) with 750 ng HBV linear DNA at 24 h post-seeding. Cells were co-transfected with 250 ng green fluorescence protein (GFP) expression vector pCMV6-AC-GFP (OriGene Technologies, MD, USA).

### 2.5. HBV Pre-Genomic RNA (pg-RNA) Analysis

Seventy-two hours post-transfection, RNA extraction, and cDNA synthesis were carried out and pg-RNA was quantified through real-time PCR as described in the transcriptome analysis section. Amplification was performed using QuantiNova SYBR Green PCR Kit (Qiagen, Hilden, Germany) with pg-RNA specific primers: Forward primer 5′-CTT TTG GAG TGT GGA TTC GC-3′ and reverse primer 5′-GCG AGG GAG TTC Quant Inova TTC TA-3′.

### 2.6. HBV DNA Quantification

#### 2.6.1. Intracellular Core-Associated DNA

Transfected cells were washed and lysed in 50 mM Tris-HCl pH 7.4, 1 mM EDTA, and 1% Nonidet P-40. Nuclei were pelleted and the supernatant was adjusted to 10 mM MgSO_4_ and treated with 100 μg/mL DNase I for 30 min at 37 °C. The reaction was stopped by the addition of EDTA at a final concentration of 25 mM [23].

#### 2.6.2. Extracellular HBV DNA

Viral particles were precipitated from culture supernatant overnight at 4 °C using 10% polyethylene glycol 8000 (PEG 8000). The precipitates were collected and re-suspended in Tris-EDTA (TE) buffer. Residual transfection DNA was removed by DNase I treatment at 37 °C [23,24]. DNA was then extracted using the QIAamp DNA Blood Mini Kit (Qiagen, Hilden, Germany).

#### 2.6.3. HBV DNA Copy Number Measurement

TaqMan Universal PCR master mix (Applied Biosystems, Thermo Fisher Scientific, MA, USA) was used as previously described [25] with minor modifications in QuantStudio 6 Flex Real-Time PCR System. Forward and reverse primers were 5′-CCG TCT GTG CCT TCT CAT CTG-3′ and 5′-AGT CCA AGA GTY CTC TTA TGY AAG ACC TT-3′, respectively, and the fluorescent probe was 5′-FAM-CCG TGT GCA CTT CGC TTC ACC TCT GC-TAMRA-3′. Serially diluted DNA plasmids, containing a single copy of full-length HBV DNA, with the identical sequence as the HBV clone used for transfection, were used as quantification standards.

### 2.7. HBV Core Antigen (HBcAg) Quantification

At 72 h post-transfection, cells were fixed and permeabilized with Cytofix/Cytoperm solution (BD Bioscience, CA, USA). Cells were incubated with mouse anti-HBcAg (Abcam, UK) followed by an Alexa Fluor 647 goat anti-mouse IgG, 2nd Ab (Invitrogen, Thermo Fisher Scientific, MA, USA). Cells were fixed in 1% paraformaldehyde prior to flow cytometry analysis using the BD LSR Fortessa. Data were analyzed using FlowJo Software (FlowJo, LLC, Oregon, USA).

### 2.8. Hepatitis B Surface Antigen (HBsAg) Analysis

Secreted HBsAg was quantified using Monolisa HBsAg ULTRA assay (Bio-Rad Laboratories, CA, USA). HBsAg levels were detected semi-quantitatively by measuring the optical density at 450 nm according to the manufacturer’s instructions.

### 2.9. Statistical Analysis

Quantitative variables were analyzed using the Student’s *t*-test. Correlation analysis was carried out using the Pearson correlation coefficient. All statistical analysis was performed using GraphPad Prism software, version 8 (San Diego, CA, USA). A *p*-value of less than 0.05 was considered statistically significant.

## 3. Results

### 3.1. Heterogeneous Gene Expression Profile in Liver Enriched TFs among Liver-Derived Cell Lines

To understand the suitability of liver-derived cell transfection models for HBV genotype B replication/transcription, we first determined the transcriptome profile of selected liver enriched TFs including the HNF family: HNF1(HNF1α, HNF1β), HNF3 (HNF3α, HNF3β, HNF3γ), HNF4 (HNF4α, HNF4γ) along with CCAAT binding protein-α (C/EBPα), peroxisome proliferator-activated receptor α (PPARα) and retinoid X receptor α (RXRα) in HCC-derived hepatoma cells and immortalized human hepatocyte cells in comparison with PHH.

**Figure 1:**
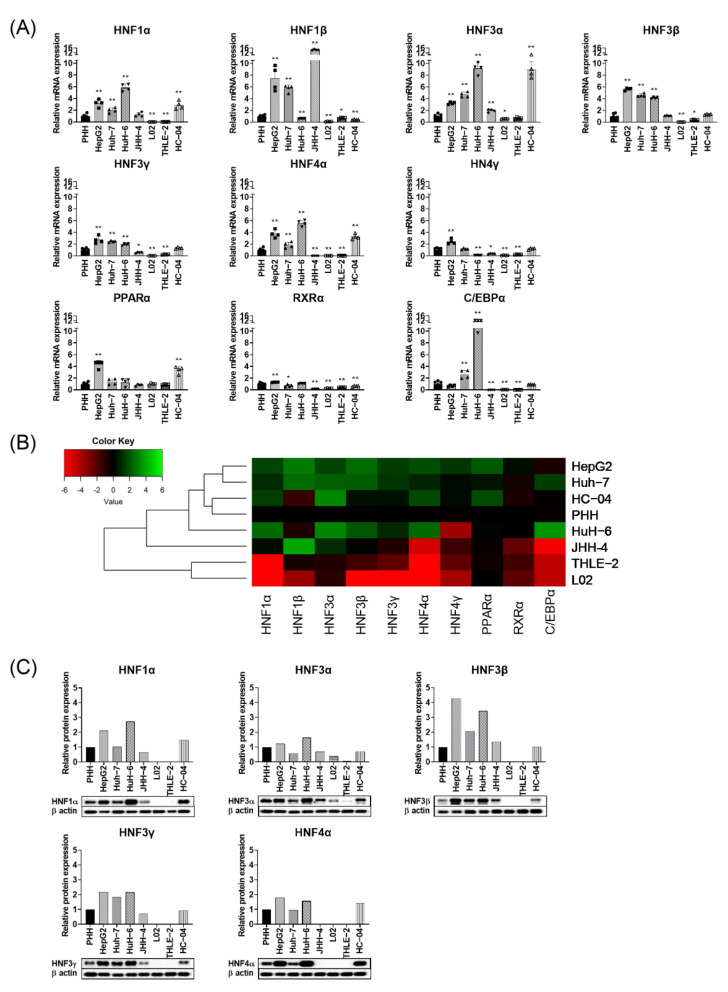
Liver enriched TFs expression level among liver-derived cell lines. (**A**) Relative mRNA expression of liver enriched TFs to primary human hepatocytes (PHH). Each data bar represents the mean + SD from two independent experiments, each done in duplicates. The values for PHH are from 3 different donors. * *p* < 0.05, ** *p* < 0.01, *t*-test done by comparing each of the cell lines to PHH. (**B**) Hierarchical clustering of mRNA expression of liver enriched TFs among hepatoma cell lines relative to PHH. (**C**) Relative protein expression of liver enriched TFs. β-actin was used as an internal control.

In the immortalized human hepatocyte line, HC-04, the majority of TFs including HNFs (HNF3β, HNF3γ, and HNF4γ), C/EBPα, and RXRα were expressed at similar levels to PHH. However, HNF3α, HNF4α, and PPARα showed 2 to 8-fold enhanced mRNA expression relative to PHH (Figure 1A).

HNF1α, HNF3β and HNF4α expression levels were enhanced 2 to 6-fold in HCC-derived HepG2, Huh-7, HuH-6, and HepG2-hNTCP cells in comparison with PHH (Figure 1A; Supplementary Materials, Figure S1A), while HNF3α and C/EBPα showed 10 to 12-fold enhanced expression in HuH-6 line (Figure 1A). The majority of studied liver enriched TFs were not expressed or expressed at significantly lower levels in L02 and THLE-2 cells (Figure 1A).

Figure 1B shows the dendrogram clustering of liver enriched TFs, which displays a close similarity between PHH and HC-04, followed by Huh-7, HepG2, and HuH-6. However, THLE-2 and L02 clustered furthest from PHH (Figure 1B).

We further investigated the protein expression of the HNF family of TF, in which enhanced mRNA expression level was observed in many cell lines (Figure 1C). Confirming our observations at the transcriptome level, HC-04 has a similar protein expression level compared to PHH (Figure 1C). Low or no expression of selected TFs was observed in L02 and THLE-2 cell lines. Among HCC-derived cell lines, HuH-6, Huh-7 and HepG2 showed a slight increase in TF protein expression relative to PHH except for HNF3β, which showed 2 to 4-fold enhanced expression in HepG2 and Huh-6 cells (Figure 1C).

These observations indicate that the cell lines studied showed heterogeneous patterns of expression of liver enriched TFs at protein and transcript levels. This differential expression level may in turn lead to differences in efficiency in supporting HBV replication/transcription.

### 3.2. Ubiquitous Transcription Factors Are Expressed at Similar Levels to PHH among Liver-Derived Cell Lines

**Figure 2:**
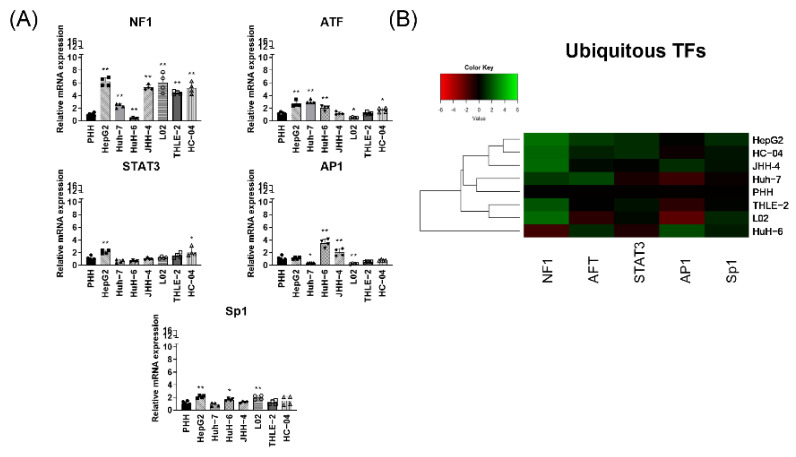
Ubiquitous TFs transcriptome profile. (**A**) Ubiquitous TFs mRNA expression quantification (relative to PHH). Each data bar represents the mean + SD from two independent experiments, done in duplicates. PHH values represent 3 donors. * *p* < 0.05, ** *p* < 0.01, *t*-test, by comparing each of the cell lines to PHH. (**B**) Hierarchical clustering of relative mRNA expression of ubiquitous TFs in liver-derived cells.

As mentioned, apart from liver enriched TFs, several ubiquitous TFs are known to play a regulatory role in HBV replication/transcription. Hence, we next investigated the mRNA expression levels of several ubiquitous TFs including NF1, activating transcription factor (ATF), STAT3, AP1, and SP1. In HC-04 cells, these were expressed at similar level relative to PHH in HC-04 (Figure 2A), except for NF1. In HCC-derived HepG2, Huh-7 and HuH-6 as well as JHH-4 cells, the ubiquitous TFs, STAT3, and SP1 were expressed at similar levels as PHH, while slight increases in ATF and AP1 were observed (Figure 2A). NF1, which plays a key role in S promoter activity, showed an enhanced expression (more than 3-fold) in all cell lines except in HuH-6. Unlike liver enriched TFs, in L02 and THLE-2 cells, ubiquitous TFs were expressed either at similar levels as PHH or enhanced by approximately 6-fold in the case of NF1 (Figure 2A). In HepG2-hNTCP cells, NF1 showed 4-fold increased expression relative to PHH while STAT3 and AP1 are expressed at similar levels as PHH (Supplementary Materials, Figure S1B).

Dendrogram clustering revealed a close similarity between Huh-7 and PHH. THLE-2 and L02 are clustered together and further from PHH while HuH-6 is clustered furthest from PHH. HC-04 and HepG2 showed more similarity in ubiquitous TF transcriptome profiles.

Unlike the heterogeneous expression profile observed in liver enriched TFs, ubiquitous TFs are expressed among all liver-derived cell lines.

### 3.3. HBV Genotype B Liver-Derived Cell Transfection Models

As mentioned, the lack of an efficient in vitro infection model, which supports all HBV genotypes infection has led to the dependence on the transfection model approach for non-D HBV genotype studies. Here, we investigated the suitability of several liver-derived cell lines for supporting HBV genotype B replication/transcription. HCC derived and immortalized human hepatocyte-derived cells were co-transfected with the full-length HBV genotype B genome and a plasmid encoding GFP ORF (for transfection efficiency assessment). We then analyzed HBV replication/transcription efficiency in each cell line and examined possible correlations with expression levels of the assessed TFs.

#### 3.3.1. HBV Replication in Liver-Derived Cell Lines Is Associated Particularly with HNF4α Expression

At 72 h post-transfection, the expression levels of viral pg-RNA, as well as the levels of HBV intracellular and extracellular DNA, were determined. These were then compared with those from HepG2.2.15 cells, which carry the HBV genotype D genome. To enable meaningful comparison, all data were normalized to the transfection efficiency of each cell line used.

**Figure 3:**
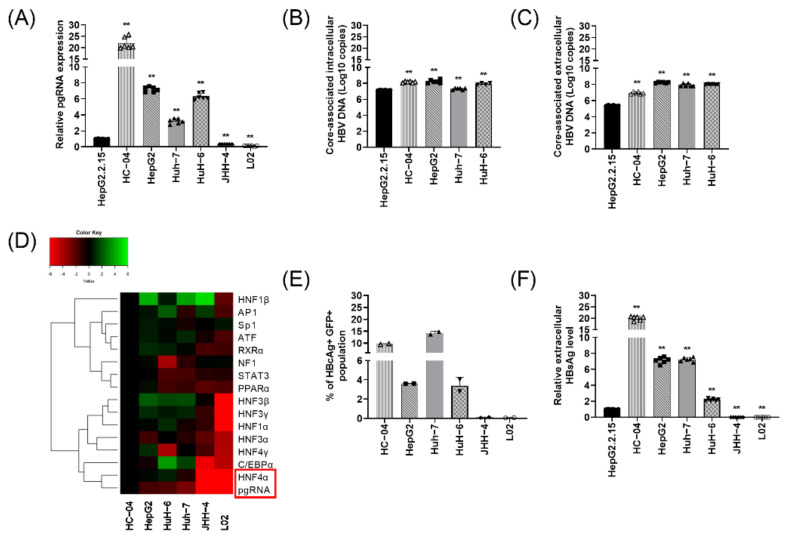
Efficiency of liver-derived cell transfection models in supporting hepatitis B virus (HBV) genotype B replication and gene expression. (**A**) Relative pg-RNA expression to HepG2.2.15 among the liver-derived cell transfection models at 72 h post-transfection. ** *p* < 0.01, T-test, by comparing each of the cell lines to HepG2.2.15. Core-associated intracellular (**B**) and extracellular HBV DNA (**C**) in cell lines with detectable pg-RNA expression level. (**D**) Hierarchy clustering analysis of relative mRNA expression of host TFs and viral pg-RNA across transfection models (**E**) The percentage of HBcAg^+^ GFP^+^ population measured by FACS analysis. (**F**) Secreted HBsAg in the supernatant of transfected cells measured by ELISA. Each data bar represents the average + SD from triplicate samples with two technical replicates. The relative HBsAg expression and *p*-value were calculated by comparing each of the cell lines to HepG2.2.15.

High quantities of pg-RNA were detected (>20-fold higher than HepG2.2.15) in HC-04 cells. pg-RNA expression was 3 to 8-fold higher than HepG2.2.15 in HCC-derived HepG2, HuH-6, and Huh-7 cells. No pg-RNA was detected in JHH-4 and L02 (Figure 3A).

Next, we measured core-associated extra- and intracellular viral DNA in cell lines with detectable pg-RNA synthesis. Despite the observed differences in pg-RNA expression level between HC-04 and HCC-derived hepatoma cells, similar levels of viral DNA were detected, that is, 6 to 8-fold higher than HepG2.2.15 (Figure 3B,C).

Hierarchical clustering was carried out to examine the correlation (if any) between pg-RNA production and the expression of host TFs. Figure 3D shows that pg-RNA is closely clustered with liver enriched TFs, specifically HNF4α, suggesting that pg-RNA expression level is highly dependent on HNF4α. Moreover, it was evident that there was closer clustering between the liver enriched TFs than between the ubiquitous TFs and pg-RNA. This is consistent with the fundamental regulatory role of liver enriched TFs in pg-RNA synthesis.

#### 3.3.2. Differential Expression of HBV Genotype B Antigens among Liver-Derived Cell Transfection Models

Multiple studies have shown that TFs regulate HBV transcription through binding to the core and preS/S promoters on cccDNA. Hence, viral core antigen and HBV surface antigen production at 72 h post-transfection were measured and compared to that of HepG2.2.15 cells.

Intracellular HBcAg expression was determined in a population of cells co-expressing HBcAg and GFP (HBcAg+GFP+) using flow cytometry analysis. Ten to fifteen percent of cells were positive for HBcAg and GFP in HC-04 and HCC-derived hepatoma Huh-7 cells (Figure 3E). Lower HBcAg expressions were observed for HuH-6 and HepG2 cells. There was no HBcAg expression in JHH-4 and L02 cells (Figure 3E).

HC-04, HepG2, Huh-7, and HuH-6 cells all showed significantly high levels of HBsAg expression after transfection when compared to HepG2.2.15 cells. The highest expression was observed in HC-04 cells with a 20-fold increase compared to HepG2.2.15 cells. As expected, HBsAg expression was not detected in JHH-4 and L02 cells (Figure 3F).

Collectively, these results suggest that some of the cell lines are not able to support the production of HBV genotype B viral antigens, and hence, are not suitable as transfection models.

## 4. Discussion

The ability of liver-derived cell transfection models to support non-D HBV genotypes and related variants’ replication/transcription is greatly influenced by the expression of transcription factors and the existence of specific TF networks.

This study unraveled the different fitness of the liver-derived cell lines for HBV genotype B replication/transcription. This is due to TFs transcriptome profile heterogeneity, with the best outcomes dependent on the degree of similarity to the profile of PHH.

Our results suggest that the HC-04 transfection model is the most competent for HBV genotype B replication and antigen production. HC-04 cells displayed the most similar liver enriched TFs transcriptome profile to PHH, indicating that this similarity is a key determining factor in efficient HBV replication and viral antigen production. Several HCC-derived cells with less similarity to PHH were also capable of supporting pg-RNA and viral antigen production, albeit at a lower efficiency compared to HC-04 cells. These include Huh-7, HepG2, and HuH-6 cells.

Our study further validates the fundamental role of HNF4α expression in HBV genotype B replication (pg-RNA synthesis). HNF4α clustered closest to pg-RNA, which suggests its expression directly controls pg-RNA synthesis. In JHH-4 and L02 models, the lack of HNF4α expression resulted in the inability of these cells to support pg-RNA and viral antigen production. Similarly, lower levels of HNF4α in the Huh-7 model led to lower pg-RNA production. Besides HNF4α expression, pg-RNA production is known to be regulated by the interaction of multiple TFs. PPARα and HNF4α interaction has been demonstrated to have an additive effect on pg-RNA synthesis, where the over-expression of PPARα and HNF4α in non-hepatic cell lines was sufficient to increase pg-RNA expression. Similarly, over-expression of PPARα in Huh-7 leads to an increased level of pg-RNA production [6,7,8]. HNF3β however, is known to decrease pg-RNA synthesis via interfering with RNA elongation [5,6]. Thus, the poor efficacy of pg-RNA production in Huh-7, HepG2, HuH-6 cells when compared to HC-04 could be due to the higher expression of HNF3β in these cells. Overall, we conclude that HC-04 efficacy in pgRNA production is the result of the augmented expression of PPARα and HNF4α, their interaction, and the absence of the inhibitory effects of HNF3β.

Our observations also show the crucial role of a specific TF network in efficient viral antigen production. In HC-04 cells, a significant increase in mRNA expression was observed only in HNF3α and NF1. Multiple studies have demonstrated that enhanced HNF3α expression positively regulates PreS2/S promoter activity [26,27]. NF1 expression has been established as an essential factor for optimal S promoter activity [6]. Considering these key regulatory roles, the presence and interaction of these TFs are important contributing factors in the optimal activity of S promoter in the HC-04 model. Taking all this into consideration, low HBsAg production detected in HuH-6, despite high HNF3α expression, could be due to lack of NF1 expression, and the consequent absence of the critical interaction of these TFs.

Overall, this study unravels the significance of the existence of a certain TFs network and the high similarity of the TFs transcriptome profile to PHH in ensuring the efficacy of the transfection model for HBV genotype B replication and gene expression. These findings could lead to a more informed choice of transfection models for studying non-D genotypes.

## Figures and Tables

**Table 1:** Characteristics of cell lines.

No.	Cell Lines	Source of the Cells	Case History	Culture Medium
1	HepG2	ATCC	HCC	DMEM supplemented with 4 mM L-glutamine, 1 mM sodium pyruvate, 10% FBS, 100 U/mL penicillin and 100 µg/mL streptomycin
2	Huh-7	RIKEN Cell Bank	HCC	Same as HepG2 cells
3	HuH-6	Japan Health Sciences Foundation	HCC	Same as HepG2 cells
4	JHH-4	Japan Health Sciences Foundation	HCC	Same as HepG2 cells
5	L02	Prof. Tao Zhu from University of Science and Technology of China	Immortalized human hepatocyte cell line	Same as HepG2 cells
6	HC-04	BEI Resources	Immortalized human hepatocyte cell line	DMEM/F12 medium (1:1) supplemented with 15 mM HEPES, 1.5 g/L sodium bicarbonate, 2.5 mM L-glutamine, 100 U/mL penicillin, 100 µg/mL streptomycin and 10% FBS
7	THLE-2	ATCC	Simian virus 40 (SV40) large T antigen-immortalized normal liver epithelial cells	BEGM BulletKit excluding GA-1000 and epinephrine aliquots with additional 5 ng/mL EGF, 70 ng/mL phosphoethanolamine, 100 U/mL penicillin, 100 µg/mL streptomycin and 10% FBS.
8	HepG2-hNTCP	Prof. Stephan Urban from University Hospital Heidelberg	HepG2 cells transduced with human sodium-taurocholate co-transporter polypeptide (hNTCP)	Same as HepG2 cells selection: 5 µg/mL puromycin
9	HepG2.2.15	[20]	HepG2 cells transfected with plasmids containing two head-to-tail dimers of the HBV genotype D genome	Same as HepG2 cells Selection: 200 µg/mL G418

DMEM: Dulbecco’s Modified Eagle’s medium; FBS: fetal bovine serum; BEGM: bronchial epithelial cell growth medium; GA: gentamycin/amphotericin; EGF: epidermal growth factor.

**Table 2:** Primers used for transcription factors (TFs).

No.	Target	Forward Primer (5′-3′)	Reverse Primer (5′-3′)	Annealing Temperature (°C)	Amplicon Size (bp)
1	HNF1α	CACGCCACCCATCCTCAAAG	CTTGACCATCTTCGCCACACG	55	118
2	HNF1β	ACAATCCACTCTCAGGAGGC	GAGGTTCCTTGTCTCCCACC	60	123
3	HNF3α	CTACTACGCAGACACGCAGGAG	GTCATGTTGCCGCTCGTAGTC	55	129
4	HNF3β	CTCACAGGCTCAACTCGGG	TCCCCTTCAGCTCTCCCAGG	60	128
5	HNF3γ	TCTGGTGACACTTCACTTGTCC	GTACCCCGTGGATCAACACC	60	103
6	HNF4α	ACGACACGTCCCCATCAGAAG	ACCGTAGTGTTTGCCCGTGG	55	107
7	HNF4γ	GGGTCAAGCACTGACATAAACG	ACAGTGCCACCTGATCATCC	60	148
8	PPARα	CGCAAACTTGGACCTGAACG	TCCATACGCTACCAGCATCCC	60	115
9	RXRα	GACGGAGCTTGTGTCCAAGAT	AGTCAGGGTTAAAGAGGACGAT	55	89
10	C/EBPα	ACAAGAACAGCAACGAGTACCG	GGTCATTGTCACTGGTCAGCTC	55	132
12	AP1/cJun	GCGCAGCCCAAACTAACCTC	AGCCATAAGGTCCGCTCTCG	60	124
13	ATF2	ACACAACTCCACAGACCCAAAG	GAAGCTGCTGCTCTATTTCGC	55	109
16	NF1	GCCTTTAACACCCGCGTACAG	TTCCAAGTCGGCTCAACTGC	55	126
18	SP1	AGTTCCAGACCGTTGATGGG	GTTTGCACCTGGTATGATCTGT	60	118
19	STAT3	CTCTACAGTGACAGCTTCCC	CCCAGGAGATTATGAAACAC	55	125

**Table 3:** Primary antibodies for Western blot.

No.	Target	Source	Manufacturer
1	HNF1α	Rabbit	Cell Signaling Technology, #89670
2	HNF3α	Rabbit	Cell Signaling Technology, #58613
3	HNF3β	Rabbit	Cell Signaling Technology, #8186
4	HNF3γ	Goat	Santa Cruz Biotechnology, sc-5361
5	HNF4α	Mouse	Abcam, ab41898
6	PPARα	Mouse	Santa Cruz Biotechnology, sc-398394
7	RXRα	Rabbit	Cell Signaling Technology, #5388
8	C/EBPα	Rabbit	Abcam, ab40764
9	β-actin	Mouse	Santa Cruz Biotechnology, sc-4778

## Data Availability

Not applicable.

## Supplementary Materials

The following are available online at https://www.mdpi.com/1999-4915/13/3/524/s1, Figure S1: Liver enriched and Ubiquitous TFs expression in HepG2-hNTCP.
