# Supplementary figures and images for: Liver-Derived Cell Transfection Model Efficacy for HBV Genotype B Replication/Transcription Is Determined by Complex Host Transcription Factor Network
Source: Viruses. 2021 Mar 22;13(3):524. doi: 10.3390/v13030524 (PMC8005026; doi:10.3390/v13030524)

(A)

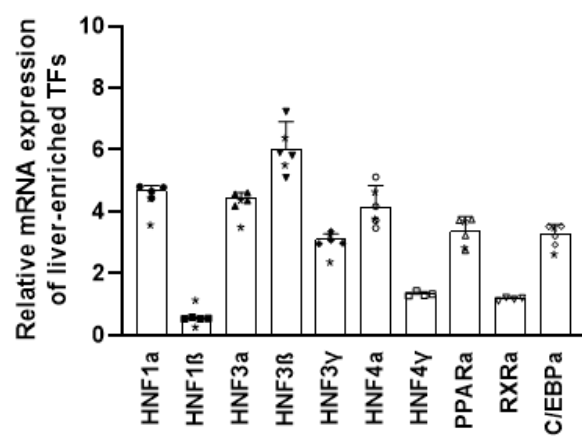

(B)

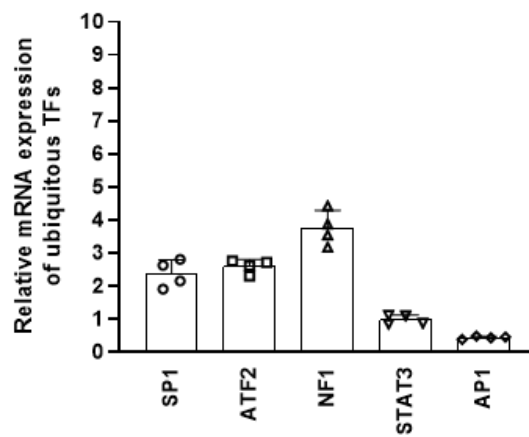

Supplement: Supplementary file 1 [file viruses-13-00524-s001.pdf]
